# Association of schistosomiasis and risk of prostate cancer development in residents of Murehwa rural community, Zimbabwe

**DOI:** 10.1186/s13027-020-00327-2

**Published:** 2020-10-06

**Authors:** Emilia T. Choto, Takafira Mduluza, Francisca Mutapi, Moses J. Chimbari

**Affiliations:** 1 University of KwaZulu Natal, School of Nursing and Public Health, 6 College of Health Sciences, Howard College, 269 Mazisi Kunene Road, Berea, Durban, 4041,7 South Africa; 2 University of Zimbabwe, Biochemistry Department, P.O. Box MP 167, Mount Pleasant, Harare, Zimbabwe; 3 University of KwaZulu Natal, School of Laboratory Medicine and Medical Sciences, Howard College, 269 Mazisi Kunene Road, Berea, Durban, 4041 South Africa; 4 Centre for Infection, Immunity and Evolution, Institute of Immunology and Infection Research, University of Edinburgh, Ashworth Laboratories, King’s Buildings, Charlotte Auerbach Road, Edinburgh, EH9 3FL UK

**Keywords:** Schistosomiasis, Male genital schistosomiasis, Prostate cancer, Prostate specific antigen, *S. haematobium*, *S. mansoni*, Urogenital schistosomiasis

## Abstract

**Background:**

Prostatic male genital schistosomiasis and prostate cancer co-existence cases are uncommon however, some studies have indicated that schistosomiasis may trigger development of prostate cancer regardless of age. Schistosomiasis is a public health problem in sub-Saharan Africa and may account for some undocumented cases of schistosomiasis prostatic cancer in schistosome endemic rural communities. It is against this background that we investigated the association between schistosomiasis and risk of prostate cancer development in residents of Murehwa Community, a schistosomiasis endemic area.

**Methodology:**

We conducted a cross sectional study involving 366 men residing in Murehwa District, Zimbabwe. *Schistosoma haematobium* and *S. mansoni* infection was diagnosed using urine filtration and Kato Katz techniques, respectively. Haematuria was detected using urinalysis reagent strip test. A structured questionnaire was used to obtain history of schistosomiasis infection among study participants. Risk of prostate cancer development was assessed by measuring prostate-specific antigen levels in serum using the ELISA.

**Results:**

Prevalence of *S. haematobium* and *S. mansoni* infection was 12.3% and 1.4%, respectively. Individuals with schistosomiasis had higher prostate-specific antigen levels (mean 1.208 ± SD 1.557 ng/mL) compared to those without schistosomiasis (mean 0.7721 ± SD 1.173 ng/mL; *p* < 0.05). Older individuals > 50 years had higher prostate specific antigen levels (mean 0.7212 ± SD 1.313 ng/mL) compared to individuals < 50 years old (mean 0.4159 ± SD 0.8622 ng/mL; *p* < 0.05). Prostate-specific antigen levels log_10_ (mean 0.2584 ± SD 0.2128 ng/mL) and were associated to *S. haematobium* infection intensity log_10_ (mean 1.121 ± SD 0.5371 eggs/10 mL), r(s) = − 0.3225, *p* < 0.05. There was a correlation between prostate-specific antigen levels log_10_ (mean 0.2246 ± SD 0.1858 ng/mL) and *S. haematobium* infection intensity log_10_ (mean 1.169 ± SD 0.5568 eggs/10 mL) among participants with a history of schistosomiasis infection (r(s) = − 0.3520; *p* < 0.05). There was no correlation between prostate-specific antigen levels of > 4 ng/mL (mean 5.324 ± SD1.568 ng/mL) and schistosome eggs log_10_ (mean 1.057 ± SD 0.6730 eggs/10 mL; *p* > 0.05).

**Conclusion:**

Urogenital schistosome infections and history of schistosome infections were associated with prostate specific antigen levels, an indicator for risk of prostate cancer. Therefore, *S. haematobium* schistosome egg burden was associated with the risk of prostate cancer development in adult males residing in Murehwa District, Zimbabwe.

## Introduction

Prostatic male genital schistosomiasis (MGS) and prostate cancer co-existence cases, have been reported since the 1980s [1] and continue to recur as recently reported by Lodhia *et. al.* (2020) [2]. Prostate cancer is a common urogenital condition living in both schistosomiasis and non-schistosomiasis endemic areas. Despite geographical locations, coincidences of MGS infections and prostate cancer cases have also been reported in non-schistosome endemic areas [3–5]. The concurrent incidences of prostatic MGS and prostate cancer have prompted the suggestion of a causal association between the two diseases.

Prostate cancer is the second most common cancer after lung cancer and the fifth leading cause of cancer-associated mortality among men worldwide [6]. More than 1 million new cases were recorded in 2018 and the disease accounts for approximately 4% deaths caused by cancer in men [7]. In Zimbabwe, prostate cancer is the most common and leading cause of death [8]. Limited existing literature on prostate cancer in Zimbabwe showed that there was major increase in prostate cancer incidences by 6.4% annually from 1991 to 2010 due to westernization of lifestyles [9]. In 2017, Zimbabwe Cancer Registry reported that prostate cancer accounts for more than 25% percent of all cancers affecting men hence a public health problem in the country [10]. More than 80% of prostate cancer cases are diagnosed very late and the disease contributes to 9% of cancer related deaths yearly to cancer [10].

The risk of prostate cancer is associated with men above 50 years, ethnicity, race, geographic region and family history of prostate cancer [11, 12]. Prostate cancer diagnosis is assessed by a serum prostate-specific antigen test and digital rectal examination [13]. Prostate-specific antigen is a kallikrein-like serine protease produced in the epithelial cells of the prostate, which is organ but not prostate cancer specific [14]. Screening for prostate cancer using prostate-specific antigen levels aims to detect prostate cancer early stage for better management of prostate cancer and reduction of disease specific mortality [15]. Elevated prostate-specific antigen levels indicate greater likelihood of prostate cancer but can also attributable to other health conditions such as prostatitis, benign hyperplasia prostatic inflammation [11, 14]. The sensitivity of a prostate-specific antigen levels above 4.0 ng/mL for detecting prostate cancer ranges from 63 to 83% hence serves as a reference point for further prostate cancer analysis [16, 17]. However, the risk of prostate cancer has been reported in relation to low prostate-specific antigen values range from 6.6 to 26.9% from prostate-specific antigen levels of < 0.5 ng/mL and 3.1 ng/mL - 4 ng/mL respectively [16]. Hence, many men may still have likelihood of prostate cancer development despite having low serum prostate-specific antigen [18]. More so, it has been speculated that men with the higher prostate specific antigen levels are more likely to harbour premalignant lesion also known as small prostate cancer that progresses to become clinically apparent decades later [19]. Some studies have shown that there is risk of prostate cancer development despite the low prostate specific antigen levels over years [19, 20]. Prostate cancer symptoms include frequent urination, nocturia, difficulty in starting and maintaining a steady stream of urine, haematuria, and dysuria [21]. Some of the symptoms such as haematuria, dysuria and nocturia are synonymous with those of schistosomiasis infection. Therefore, men that reside in schistosomiasis endemic areas may confuse prostate cancer symptoms with those of schistosomiasis.

Schistosomiasis is a disease caused by the trematodes of different species of the genus *Schistosoma* [22]. The disease causes irreversible damages to certain body organs such as the bladder and the liver and accounts for the high burden of morbidity in areas where it is endemic [23]. More than 700 million people are at risk of contracting schistosome infection, with 200 million of them residing in sub-Saharan Africa [24–26] .An estimated 95.2 million adults are at risk of schistosomiasis infection [23]. *Schistosoma haematobium* and *Schistosoma mansoni* are the two species endemic in Zimbabwe. Although *S. haematobium* is relatively less important as a cause of mortality compared to *S. mansoni*, its effects can trigger other diseases such as cancer and its combination with other diseases such as HIV and cancer may result in high mortality [23]. *S. haematobium* adult worms produce hundreds to thousands of ova daily [27]. The eggs are passed out through urine but some of the ova penetrate and lodge in the vessel walls of nearby tissues mainly in the bladder, bowel and to a lesser extent the seminal vesicles, vas deferens, prostate gland, spermatic cord and the penis resulting in MGS [28, 29]. Whilst, *S. mansoni* paired adult trematodes that reside in the peripheral vessels of the intestines causing intestinal schistosomiasis [22].

Prostatic MGS is uncommon however; past or active schistosome ova were identified in the prostate and seminal vesicles of approximately 15% of all males between the ages of 3 months to 70 years in an autopsy study done by Cohen *et. al*. in 1993 in Southern Africa [30]. Additionally, in 1970, Gelfand *et. al*. in Zimbabwe reported of approximately 21% of the cadavers having schistosome eggs in the prostate [31]. In response to the embedded schistosome eggs, the body will orchestrate granuloma lesions formation due to inflammation in the genitals causing genital lesions [32]. The genital lesions may lead to events that could indirectly lead to carcinogenesis. *S. haematobium* and *S. mansoni* associated prostate adenocarcinoma cases has been reported in different schistosomiasis endemic countries such as Angola, Egypt, South Africa and Brazil and also schistosomiasis non endemic areas such as USA and Iraq [33–36]. Thus available literature, though very limited, suggests a possible association of schistosomiasis infection with the development of prostate cancer particularly in schistosomiasis endemic areas.

One of the 10 provinces in Zimbabwe, Mashonaland East, has the second highest schistosomiasis prevalence amongst school going aged children ranging from 9.0 to 53.0% across the province [37]. Approximately 13% of the number of all cancer patients reported in Zimbabwe in 2015 were from Murehwa District, Mashonaland East [8]. This is the highest number of registered cancer patients in any province after the two-referral cities in Zimbabwe, Harare (35%) and Bulawayo (16%) [8]. Therefore, Murehwa District served as the ideal study area to assess the association of the two diseases. Men that reside in schistosome endemic areas have constant exposure to schistosome infections and may have active or history of MGS infections that could indirectly lead to prostate carcinogenesis. We therefore, conducted this study to determine the prevalence of schistosomiasis and association between schistosomiasis and risk of prostate cancer development in residents of a schistosomiasis endemic area.

## Methods and materials

### Study design and study area

**Fig. 1:**
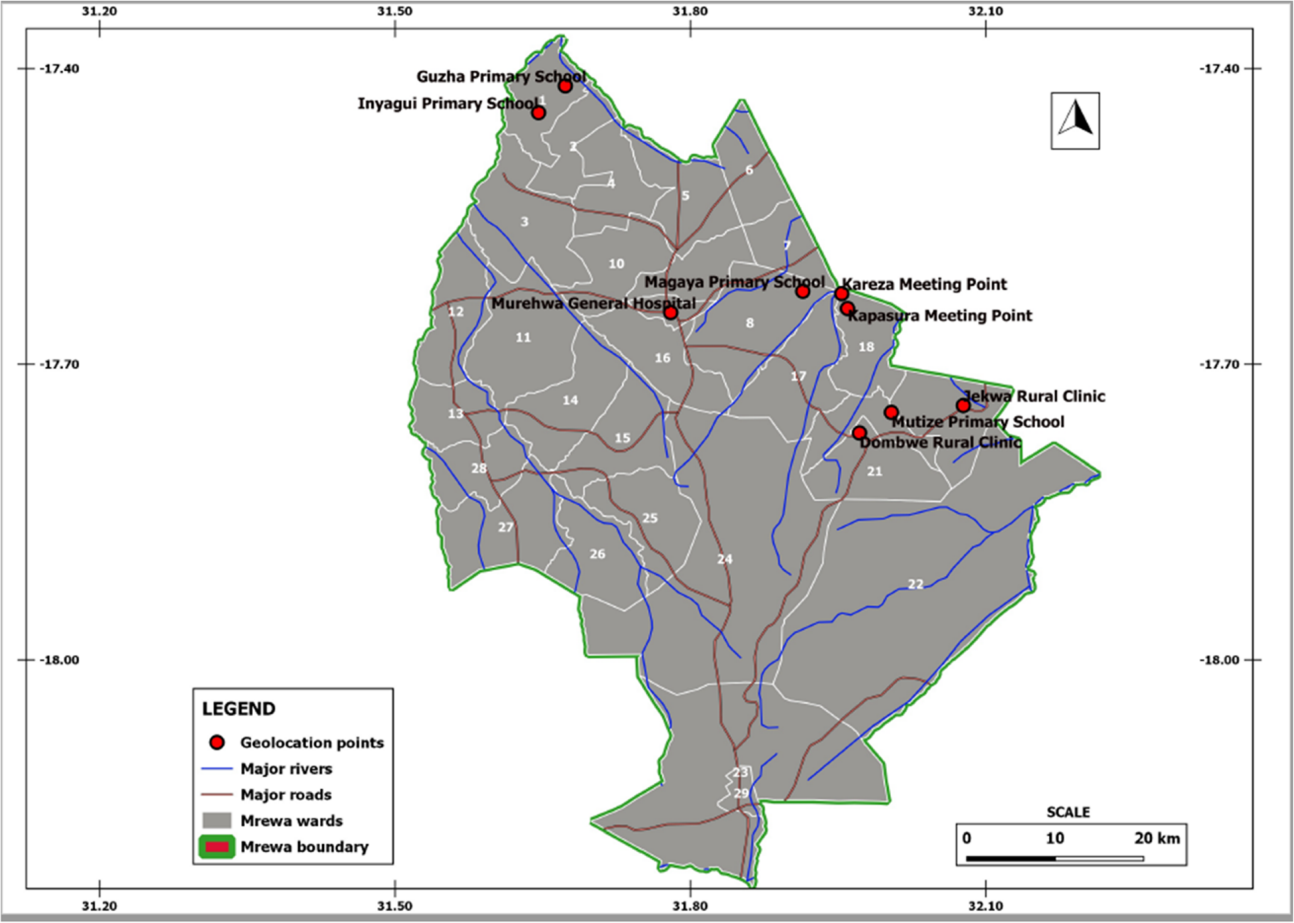
Map of Murehwa District, Mashonaland East Province, Zimbabwe and study focal meeting points

A cross sectional study was conducted in Murehwa District in 2019, targeting adult males from different villages (Kapasura, Magaya, Guzha, Dombwe, Mutize, Kareza, Inyagui and Jekwa). Murehwa is located in the Mashonaland East Province and its geographical coordinates are 17°38′35.59“ S 31°47’2.40” (Fig. 1). The overall population of Murehwa District is 199,607 and 47% are males [38]. The District consists of more than 90% of the area is rural areas. It is wet season is warm and mostly cloudy and the dry season is comfortable and mostly clear. There is poor sanitation and some of the residents practise open defecation.

### Sample size calculation and sampling

A sample size of 245 including 20% add on to account for drop out was calculated based on the inferred schistosomiasis prevalence for adults of 15.8% (a third of the 47.4% for the school aged children) [37]. We purposely recruited male adults aged 18 years and above residing Murehwa District, Zimbabwe. However, the number of participants recruited ended up being 366 because more men who met the study criteria. The study inclusion criteria were as follows: i) 18 years and above, ii) having lived in the study area all their life iii) with or without schistosomiasis iv) with or without prostate cancer and v) gave consent to be in the study during baseline. The exclusion criteria were as follows: i) aged of less than 18 years and below, ii) did not give consent to be in the study.

### Parasitological diagnosis

Urine samples of approximately 50 mL were collected from each study participant on three consecutive days between 10:00 h and 14:00 h, a period when there is peak number of eggs in the urine. The urine samples were processed and examined using the urine filtration technique [39]. The number of eggs were expressed per 10 mL of urine. Each urine sample was tested for haematuria by dipping the urinalysis reagent strip ends (Spinreact, SA lot URS 7100133A) into urine for at least 1 min. The test area for micro-haematuria was compared with a standard chart as per manufacturer’s instructions. The strength of the colour change indicates varying concentrations of blood present in the sample ranging from negative, trace (±), light (+), moderate (++) and heavy (+++).

Stool samples also collected over 3 days, processed, and examined using the Kato–Katz method [40]. The *S. mansoni* eggs were expressed per gram of stool. Participants were diagnosed positive for schistosomiasis infection if any of the two species of the parasite egg was detected in their urine or stool samples. Infection intensity was classified in accordance with the World Health Organization (WHO) guidelines as follows: for *S. haematobium*, no infections 0 eggs/10 mL, light infection ≤10 eggs/10 mL and heavy infection ≥50 eggs/10 mL and for *S. mansoni*, light infections < 100 eggs per gram (epg), moderate infections ≥100 < 400 epg and heavy infections ≥400 epg [41]. Participants who tested positive for schistosomiasis by egg count for any of the species were treated with praziquantel (PZQ) at the standard dose of 40 mg/kg per body weight.

### History of schistosomiasis infection characterisation

A structured questionnaire designed to extract the following information was administered to all study participants: past/history of schistosome infection, treatment of previous schistosomiasis infection, currently suspecting if they have schistosomiasis infection, blood in urine, pain during urination, felt pain in their genital area and if they have been screened for prostate cancer before.

### Determination of prostate specific antigen

Serum was obtained from the venous blood into blood collecting tubes without anti-coagulant (BD vacutainers Lot- 7,114,655). Qualitative detection of prostate-specific antigen levels was done using enzyme linked immuno-sorbent assay (ELISA) using the R &D Human Kallikrein 3/prostate-specific antigen Duo Set ELISA; DY1344 and R&D systems catalog # DY008 ancillary kit (96 well microplates, plate sealers, substrate solution, stop solution, plate coating buffer (PBS), wash buffer, and reagent diluent concentrate) according to the manufacturer’s instruction. All samples and standards were measured in duplicate and concentrations were determined from a standard curve using mean optical density values. Serum prostate-specific antigen concentrations were expressed as ng/mL.

### Statistical methods

Data was analysed using graph pad prism version 6.0 and Statistical Package for Social Sciences (SPSS) statistics version 16. Descriptive statistics were applied on the following variables; age, infection status and infection intensity. Mann Whitney test was used to determine the differences of prostate-specific antigen infection statuses: schistosomiasis positive and negative, haematuria positive and haematuria negative and those with a history of schistosomiasis and those without. Kruskall Wallis test was used to determine differences of schistosomiasis infection intensities. Multivariable logistic regression was used to estimate the association of schistosomiasis infection status, history of schistosomiasis and haematuria intensity with schistosomiasis infection status. Spearman’s rank correlation (Spearman’s rho) was used to determine association between schistosomiasis infections and prostate cancer development and schistosomiasis infections and hemoglobin levels. Multiple regression model using schistosomiasis infection status, history of schistosomiasis and haematuria intensity was used to predict prostate-specific antigen levels > 0 ng/mL (indicator for risk of prostate cancer development). *P* values < 0.05 were considered significant. The mean and the standard deviation were used in reporting data values.

## Results

In total, 366 individuals aged 18 to 95 years participated in the study. Twelve percent (12.3%) were infected with *S. haematobium* and 1.4% had *S. mansoni,* giving an overall schistosomiasis infection prevalence of 13.7%. Of the 45 men infected with *S. haematobium*, 19 (42%) had light infections and 26 (58%) had heavy infections. For those infected with *S. mansoni*, 1 participant with 120 epg (20%) had moderate infection intensity and 4 (80%) had light infections with 20 ± 12 epg. Kapasura community had the highest schistosomiasis infection (37.74%) followed by Kareza (13.51%), Magaya (13.18%), Inyagui (11.9%), Guzha (10%), Jekwa (3.45%) and Dombwe (3.22%). No schistosome infections were detected in participants from Mutize village. Characteristics of the participants by villages are shown in Table 1. Table 2 shows that infection intensity was significantly higher in the < 50 years age group (0.1902 ± 0.4580 log_10_ mean eggs/10 mL + 1) compared to the > 50 years age group (0.07755 ± 0.3182 log_10_ mean eggs/10 mL + 1; *p* < 0.05). Of the 50 positive for schistosomiasis infection, 18 were haematuria positive and 31 were negative. Seventeen of the 316 participants who tested negative for schistosomiasis infection were positive for haematuria. Haematuria intensity among the 35 participants were as follows: 3 (8.6%) showed 5–10 mark, 6 (17.1%) showed trace ±, 2 (5.7%) showed +, 7 (20%) showed ++ and 17 (48.6%) showed +++. Logistic regression was used to predict the likelihood of the participants to have schistosomiasis infection. The predictor values were history of schistosomiasis infection, haematuria status, villages and age. The model was statistically significant *X*^*2*^ (10, *n* = 325) = 71.420 *p* < 0.001. The model explained 36.4% (Nagelkerke R [2]) of the variance in schistosomiasis infection and correctly classified 89.5% of the cases. Haematuria status *p* < 0.001, age *p* = 0.017 and all villages *p* = 0.004 contributed significantly to the model, but history of schistosomiasis infection (*p* = 0.268) and villages (*p* > 0.05) did not contribute significantly to the model (Table 3**)**. Participants in Kapasura village (OR 3.220; 95% C.I. 0.918–11.3, *p* > 0.05) were more likely to have schistosomiasis infection than the other villages (Magaya, Kareza, Inyagui, Guzha, Jekwa, Dombwe and Mutize). History of schistosomiasis infection was associated with having schistosomiasis infection (OR 0.594; C.I. 0.211–1.67, *p* > 0.05).

**Table 1:** Number of participants and schistosomiasis prevalence in each community

Site	Number of participants	***S. haematobium*** infected	***S. mansoni*** infected	Schistosomiasis prevalence
**Mutize**	23	0	0	0.00%
**Dombwe**	31	1	0	3.22%
**Jekwa**	29	1	0	3.45%
**Guzha**	60	6	0	10.0%
**Inyagui**	42	4	1	11.9%
**Magaya**	91	11	1	13.18%
**Kareza**	37	3	2	13.51%
**Kapasura**	53	19	1	37.74%
**Total**	**366**	**45**	**5**	**13.66%**

**Table 2:** Prevalence and intensity of schistosomiasis by age group

Age group	n (%)	Schistosomiasis infected n (%)	Mean schistosome egg count (log_**10**_ ± SD)	***p*** Value (Mann-Whitney)
< 50 years	216 (59.0)	40 (10.9)	0.1902 ± 0.4580	**0.002**
> 50 years	146 (39.9)	9 (2.5)	0.07755 ± 0.3182	
Missing ages	4 (1.1)	0	0	
**Total**	366 (100)	49 (13.4)

**Table 3:** Multivariable logistic regression predicting decision from history of schistosomiasis infection, haematuria status, villages and age

	Coefficient	Wald ***X***^***2***^	***P***	Odds Ratio (95.0% C.I.)
Haematuria Status	−2.723	30.153	**0.001**	0.066 (0.025–0.174)
History of schistosomiasis infection	−.574	1.225	0.268	0.563 (0.204–1.556)
Villages		20.762	**0.004**	
Dombwe	−1.475	1.455	0.228	0.229 (0.021–2.515)
Jekwa	− 1.875	2.334	0.127	0.153 (0.014–1.7)
Mutize	−18.262	.000	0.998	0.000 (0.00)
Kapasura	1.169	3.333	0.068	3.220 (0.918–11.298)
Kareza	−.657	.550	0.458	0.519 (0.091–2.942)
Magaya	−.346	.270	0.604	0.708 (0.192–2.611)
Guzha	−1.147	2.106	0.147	0.318 (0.067–1.495)
Age	−.029	5.651	**0.017**	0.972 (0.949–0.995)
Constant	1.960	5.121	**0.024**	

### Structured questionnaire analysis

Data from the questionnaire is summarized in Table 4**.** Eighty-six of the participants suspected they had schistosomiasis infection because they experienced pain during urination, noticed blood in their urine and were frequently urinating. Two hundred and thirty (68%) of the participants had schistosome infections during their younger ages and 33 (13.9%) had current schistosomiasis infection. Two hundred of the participants had schistosomiasis infection between the ages of 1 to 20 years. One hundred and eighty three participants (77.5%) were at some point treated for schistosomiasis infection, 36 (15.3%) had never been treated and 17 (7%) could not recall having been treated for schistosomiasis. Thirty-five participants (10.6%) were noticing blood in their urine, 104 (32.6%) felt pain during urination and 88 (27.5%) felt pain in their genital area. Three hundred and fifteen participants (92.6%) had never been screened for prostate cancer and of the 10 that had been screened only 2 have confirmed prostate cancer. Of the 2 prostate cancer diagnosed participants, one was a 69 year old man who resided in Inyagui village, with a history of schistosomias infection during his teenage years which he was treated for using traditional methods. He had no active schistosome infection and he suspected he had schistosomiasis infection because he felt pain during urination however he neither had active schistosome infection nor haematuria. The second participant from Mutize village in his 60s diagnosed with prostate cancer had history of schistosomiasis infection during his teenage years and he was not treated for the infection. He did not have active schistosomiasis infection.

**Table 4:** Structured Questionnaire Responses

Parameter	Yes % (n)	No % (n)
History of schistosomiasis infection	68 (230)	32 (108)
Treated for schistosomiasis infection	77.5 (183)	15.3 (36)
Currently suspected of schistosomiasis infection	25.8 (86)	74.2 (247)
Noticed blood in their urine	10.6 (35)	89.4 (294)
Felt pain during urination	32.6 (104)	67.4 (215)
Feel pain in the genital area	27.5 (88)	72.2 (231)
Screened for prostate cancer	3.1 (10)	92.6 (315)

### Prostate specific antigen levels analysis

**Fig. 2:**
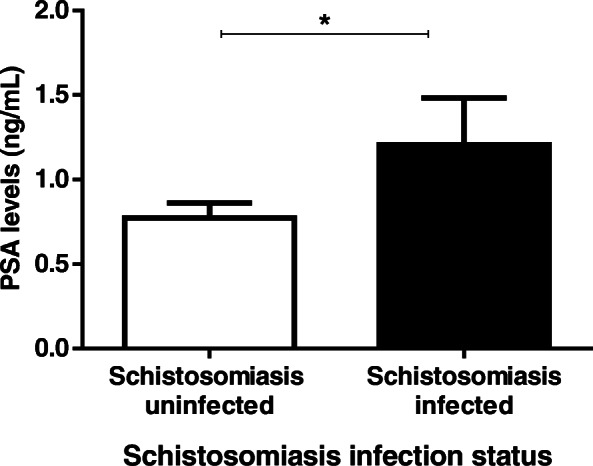
Prostate-specific antigen levels according to schistosomiasis infection, haematuria status and history of infection status

**Fig. 3:**
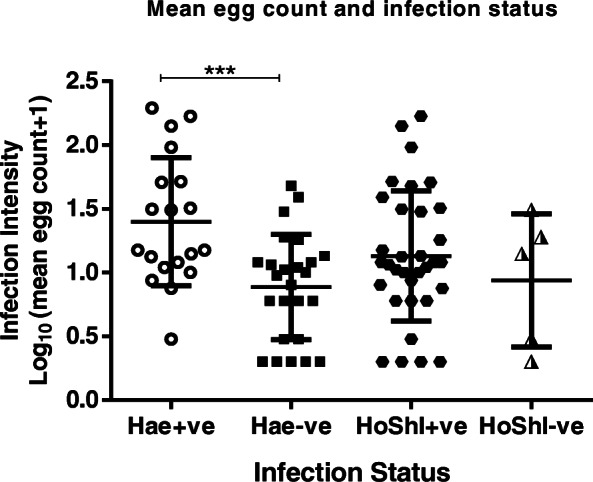
Infection intensity according to haematuria and history of schistosomiasis. HoShI - history of schistosomiasis infection, Hae - haematuria, −ve - negative, +ve - positive

**Fig. 4:**
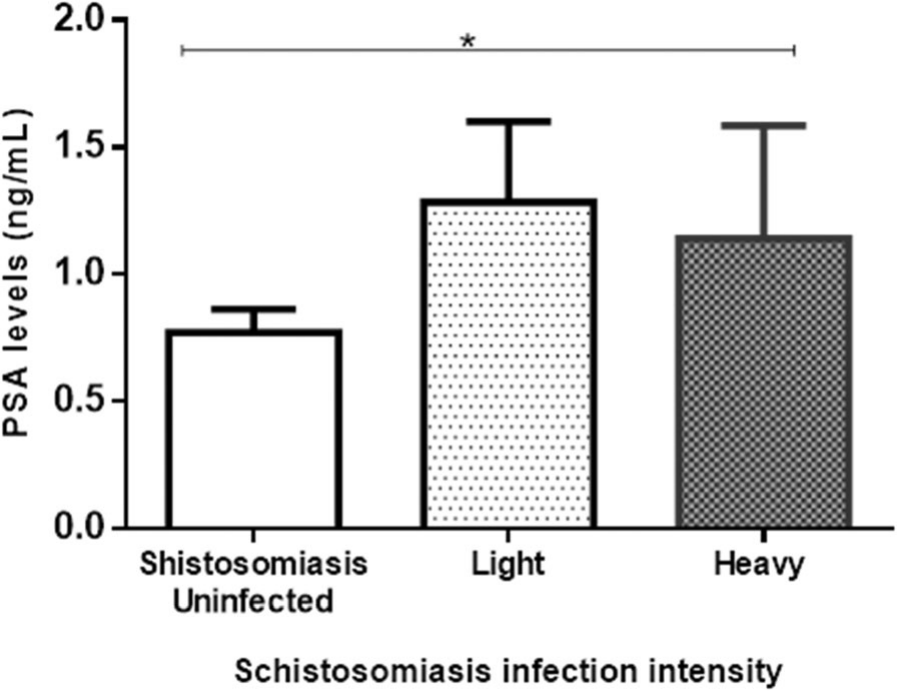
Prostate-specific antigen levels differences and schistosomiasis infection intensity

**Fig. 5:**
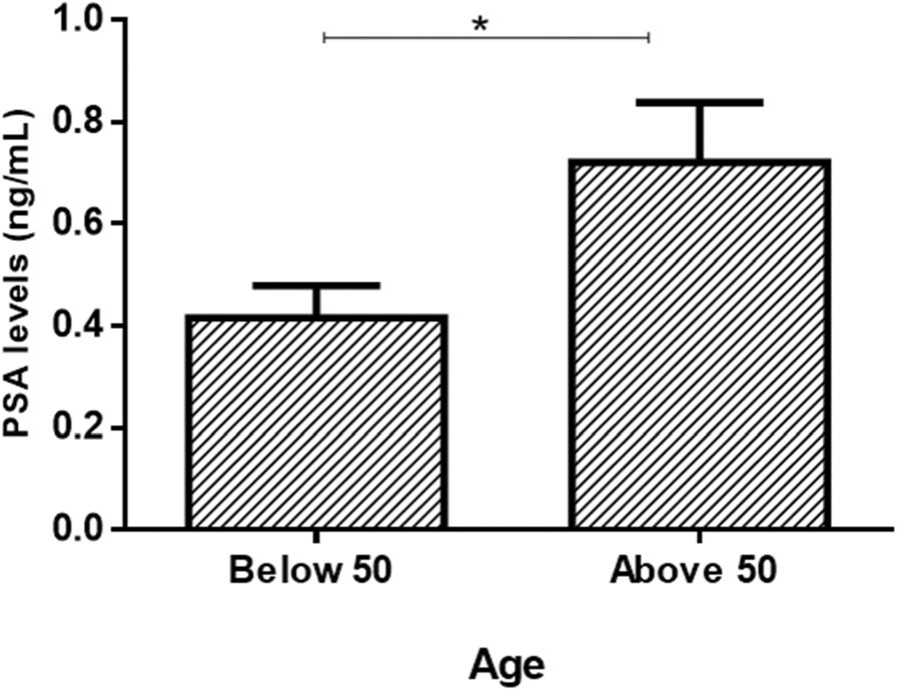
Prostate-specific antigen levels differences by age groups (> 50 and < 50)

Of the three hundred and sixty six participants, 198 (166 schistosomiasis negative and 32 schistosomiasis positive) had detectable prostate-specific antigen levels > 0 ng/mL ranged from 0.118 to 8.100 ng/mL with a mean of 0.8425 ± SD 1.249 ng/mL. Eight participants had prostate-specific antigen levels > 4 ng/mL and 5 of the 8 participants had history of schistosome infections during their younger ages. Spearman’s rank-order correlation showed no correlation between prostate-specific antigen levels of > 4 ng/mL (5.324 ± 1.568) and log_10_ mean egg + 1 (1.057 ± 0.6730; *p* = 0.333). Prostate-specific antigen levels among the schistosomiasis infected group (1.208 ± 1.557 ng/mL) were significantly higher than for the schistosomiasis uninfected group (0.7721 ± 1.173 ng/mL; *p* = 0.0221) as shown in Fig. 2. Prostate-specific antigen levels of participants without haematuria were higher (0.8740 ± 1.308 ng/mL) but not statistically significant than for those with haematuria (0.5763 ± 0.478 ng/mL; *p* > 0.05. Similarly prostate-specific antigen levels for participants without history of schistosomiasis infection (1.184 ± 1.768 ng/mL) were not significantly higher than for those with history of schistosomiasis infection (0.7882 ± 1.090 ng/mL; *p* > 0.05). Log_10_ mean egg counts + 1 of individuals that were positive for schistosomiasis and haematuria (1.400 ± 0.5038 eggs/10 mL) were significantly higher (Fig. 3) than schistosomiasis positive and but haematuria negative group (0.8859 ± 0.4128 eggs/10 mL; *p* = 0.0009). However, there was non-significant higher mean eggs for those with a history of schistosomiasis (1.130 ± 0.5103 eggs/10 mL) compared to those without history of schistosomiasis (0.9389 ± 0.5205 eggs/10 mL; *p* = 0.1183). Prostate-specific antigen levels in participants with both *S. mansoni* and *S. haematobium* light infection intensity group (*n* = 15; 0.7721 ± 1.173 ng/mL) was significantly higher compared to the heavily infected (*n* = 17; 1.141 ± 1.833 ng/mL) and schistosome uninfected infection status (*p* = 0.0341) as shown in Fig. 4. Furthermore, there was non-significant higher prostate specific-antigen levels among the *S. mansoni* (*n* = 3; 2.275 ± 2.072) infected participants compared to *S. haematobium* (*n* = 32; 1.0970 ± 1.498) infected (*p* = 0.1105). Participants older than 50 years had significantly higher prostate specific-antigen levels (0.7212 ± 1.313 ng/mL) compared to participants < 50 years old (0.4159 ± 0.8622 ng/mL; p (0.0140) < 0.05 **(**Fig. 5**)**.

Multiple regression analysis was run to predict prostate-specific antigen levels > 0 ng/mL as an indicator for risk of prostate cancer development from schistosomiasis infection status, history of schistosomiasis infection and haematuria intensity predicted prostate-specific antigen levels > 0 ng/mL F (3, 184) = 3.872, P (0.010) < 0.05, R^2^ = 0.059. Two variables, schistosomiasis infection status p (0.07) < 0.05 and haematuria intensity *p* = (0.026) < 0.05) contributed significantly to the prediction model. The highest contributing predictor was the schistosomiasis infection status (β = 216) followed by haematuria intensity (β = − 177) to explain prostate-specific antigen levels. History of schistosomiasis infection was not a significant predictor to the model (p (0.056) > 0.05) and hence did not contribute in explaining prostate-specific antigen levels > 0 ng/mL, when the other two significant predictors are in the model.

**Fig. 6:**
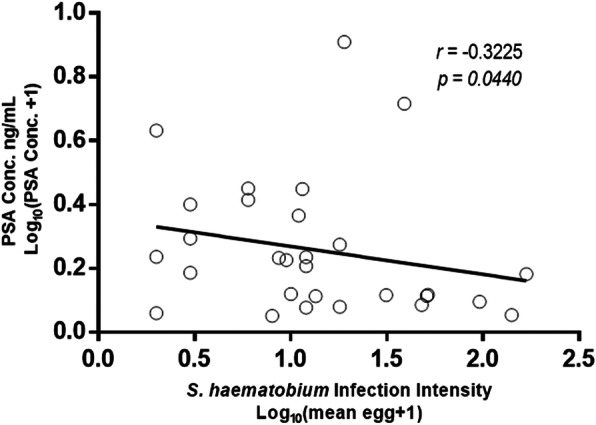
Prostate-specific antigen levels relationship with schistosomiasis infection intensity

**Fig. 7:**
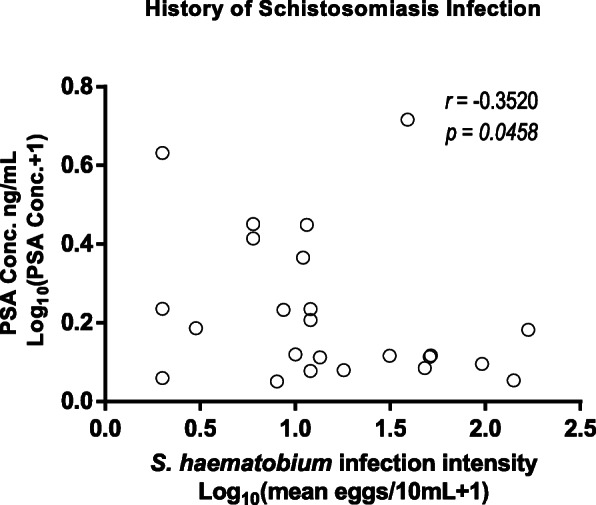
Prostate-specific antigen levels relationship with schistosomiasis infection intensity of those with a history of schistosomiasis

Of the 45 participants with *S. haematobium* infection 29 had detectable mean log_10_ (egg count+ 1); prostate-specific antigen levels of 0.2584 ± 0.2128 ng/mL and log_10_ (mean egg count + 1); 1.121 ± 0.5371 eggs/10 mL. A Spearman’s rank-order correlation showed an inverse correlation between prostate-specific antigen levels and infection intensity of participants that had schistosomiasis infection (*p* < 0.05; Fig. 6). Similarly as shown in Fig. 7, there was a negative correlation between log_10_ (egg count + 1) prostate-specific antigen levels log_10_ (0.2246 ± 0.1858 ng/mL) and *S. haematobium* mean egg counts (1.169 ± 0.5568 eggs/10 mL) among 24 participants with a history and active schistosome infections (*p* < 0.05).

## Discussion

Schistosomiasis induced prostatic cancer is not yet well characterised in populations living in schistosomiasis endemic countries to at least establish the association of male genital schistosomiasis with prostate cancer development. The main aim of this study was to determine the association of schistosomiasis infections on prostate cancer development among individuals residing in schistosomiasis endemic rural community. We found an association between prostate-specific antigen levels and schistosomiasis infection status among the study population. Additionally, we found some significant association between prostate-specific antigen levels of individuals with schistosomiasis infection having history of schistosomiasis infection with schistosomiasis infection intensity. In contrast to Elfaki *et. al*. in 2015, they did not find detectable prostate-specific antigen levels in serum samples from individuals that had schistosomiasis infection [21] and concluded that there was no association between schistosomiasis and prostate cancer. Even though we detected prostate-specific antigen levels in individuals with schistosomiasis, we cannot infer an association of the disease with prostate cancer development because prostate-specific antigen levels serve as reference points for further prostate cancer analysis and not definite prostate cancer hence further examinations such as biopsy is required. Moreover, there was no association between prostate-specific antigen levels greater than 4 ng/mL and schistosomiasis infection intensity. Therefore, we could not conclusively establish the association of schistosomiasis infection and a greater likelihood risk of prostate cancer development probably due to only 3 participants with schistosomiasis and prostate-specific antigen levels > 4 ng/mL. Interestingly, a study done by Catalona *et. al.* revealed prostate malignancy in 33% of 112 men with a prostate specific antigen levels ≥4 μg/L showing low specificity of prostate specific antigen elevated levels on prostate cancer diagnosis [42].

The elevated prostate-specific antigen levels we observed could have be attributed to old age, prostatitis, benign hyperplasia, urinary tract infections, ejaculation, prostate injury and medicine prolonged bicycles rides [43]. Prostatic inflammation due to *S. haematobium* or *S. mansoni* ova deposits can lead to elevated levels of systemic prostate-specific antigen levels [5, 44, 45]. Furthermore, other studies that reported prostatic schistosomiasis cases with high prostate-specific antigen levels were not associated with prostatic schistosomiasis cancer [44, 45]. Other studies have reported mixed outcomes whereby, men with prostate-specific antigen levels below 4.0 ng/mL had prostate cancer and on the other hand, men with higher levels of prostate-specific antigen did not have prostate cancer [18]. The presence of prostatic inflammation may disrupt the prostatic environment with time and may result in elevated prostate specific antigen levels and a greater risk of prostate cancer development [19]. Additionally, inflammation has been implicated to result to oxidative stress that could be a risk for prostate cancer development [46]. In view of this and the evidence we provided on association between history schistosome infections with active schistosome infections and risk of prostate cancer we recommend that in schistosomiasis endemic areas men with both elevated and low prostate-specific antigen levels should undergo further tests for prostate cancer.

The higher prostate-specific antigen levels detected among the schistosomiasis positive group compared to the schistosomiasis negative group could be due to schistosomiasis infection and not necessarily an indication of prostate cancer development; that could also be due to prostatic inflammation due to MGS [47]. However, prostate-specific antigen levels may not be due to schistosome infections but other conditions mentioned above. This could be because of higher prostate-specific antigen levels detected in the schistosomiasis negative group compared to the schistosomiasis severe intensity group. The higher the prostate-specific antigen level value, the higher the likelihood risk of prostate cancer [11] and the lower the prostate-specific antigen value the less likely the risk of prostate cancer. Risk of prostate cancer has been reported in relation to low prostate-specific antigen values range of 6.6 -26.9% from prostate-specific antigen levels < 0.5 ng/mL and 3.1 ng/mL - 4 ng/mL respectively [11]. Also men with low prostate specific antigen levels were reported to reflect higher risk of prostate cancer development [19, 20]. This suggests that majority of the participants in the study area with low prostate specific antigen levels might have a likelihood to develop prostate cancer and hence they may need further prostate cancer assessments.

On the contrary to observations of prostatic schistosomiasis infection progressing over time as represented by a patient with prostate adenocarcinoma and high prostate-specific antigen levels (11.59 ng/mL) reported by Metrogos *et. al* in 2017 [36], we observed insignificant lower prostate-specific antigen levels of among participants with history of schistosomiasis infection compared to those without history of schistosomiasis infection. Therefore, history of schistosomiasis infection may not necessarily an indicator of prostate cancer development. Moreover, Sharma *et. al*. in 2015 reported of a patient with benign prostatic hyperplasia with *S. haematobium* prostatic infestation and low prostate-specific antigen levels [48] indicating that prostatic MGS might not result to prostate cancer over time. However, men with history of schistosome infections could be continuously suffering or will suffer from complications of the disease such as cancer development in the future.

Prostate cancer has been diagnosed in very few men who are younger than 50 years compared to men aged > 50 [49]. Despite lower prostate-specific antigen levels in participants with < 50 years old compared to men > 50 years, men with prostate-specific antigen > 1 ng/mL at 40 years were reported to be at increased risk of being diagnosed with advanced or metastatic prostate cancer [50]. Moreover, unusual 3 cases of young males below the age of 30 were reported to have schistosomiasis prostatic adenocarcinoma [30]. It is therefore possible that men with history of schistosome infections and residing in schistosomiasis endemic area could be asymptomatically suffering from schistosome complications and may have a higher risk of having prostate cancer regardless of their age.

Current data on *S. haematobium* and *S. mansoni* epidemiology in Zimbabwe is from school-aged children [37]. We found that the overall prevalence of schistosomiasis was 13.7% in adult male individuals in the study area. Haematuria status, age and villages were significant predictors of schistosomiasis infection status. This showed that schistosomiasis is prevalent not just in children who are the main targets for mass drug administration, but also in the adult males. Although haematuria is known to be a predictor of schistosomiasis infection [51], in adults it might account for other infections such as sexually transmitted diseases. Kapasura village residents were 3.2 times more likely to have schistosomiasis infection compared to the other villages probably because of their involvement in activities such as cattle herding, fishing and gardening that constantly get them in contact with cercariae infected water sources at nearby river. Distressingly, approximately 3% of the participants have had the opportunity to be screened for prostate cancer hence there is a need for regular prostate cancer screening in the study area. Lack of resources and limited health centres with prostate cancer testing facilities in the area means many men are not aware of their prostate cancer status. Concurrent existence of prostate cancer and *Schistosoma* ova have been reported in very limited cases reports, however, the relationship of the two diseases still remains poorly understood.

## Conclusion

The association of schistosomiasis and risk of prostate cancer amongst adult males shows to be more prominent in schistosome endemic area of Murehwa District. However, since existing measurement can only detect inflammation due to egg invasion more is required to further characterize schistosomiasis prostatic carcinoma development. We found an association between urogenital schistosome infections, history of schistosomiasis infection and prostate-specific antigen levels, while prostate-specific antigen levels > 4 ng/mL were not associated with current schistosome infections. We conclude that *S. haematobium* egg burden in adult males was associated with the risk of prostate cancer development residing in Murehwa District, Zimbabwe.

### Limitations

We did not have bigger sample size to fully represent the study area would include both *S. haematobium* and *S. mansoni* infections and confirmed prostate cancer diagnosed cases to provide more evidence on the differences on the association of one of the two schistosome species with prostate cancer development. Prostate biopsy was not done to confirm and determine the magnitude of prostatic schistosomiasis and prostate cancer diagnosis hence, we could only determine risk of prostate cancer.

## Data Availability

The data generated and analyzed during the current study are available from the corresponding author on a reasonable request.

## Abbreviations

MGS: Male genital schistosomiasis
PCa: Prostate cancer
PSA: Prostate-specific antigen
